# Investigating Perampanel Antiepileptic Drug by DFT Calculations and SERS with Custom Spinning Cell

**DOI:** 10.3390/molecules28165968

**Published:** 2023-08-09

**Authors:** Nicolò Simone Villa, Chiara Picarelli, Federica Iacoe, Chiara Giuseppina Zanchi, Paolo M. Ossi, Andrea Lucotti, Matteo Tommasini

**Affiliations:** 1 Department of Chemistry, Materials, and Chemical Engineering “G. Natta”, Politecnico di Milano, Piazza Leonardo da Vinci 32, 20133 Milano, Italy; nicolosimone.villa@polimi.it (N.S.V.); chiara.picarelli@polimi.it (C.P.); chiara.zanchi@tiscali.it (C.G.Z.); andrea.lucotti@polimi.it (A.L.); 2 Department of Energy, Politecnico di Milano, Piazza Leonardo da Vinci 32, 20133 Milano, Italy; paolo.ossi@polimi.it

**Keywords:** quantitative SERS, spinning cell, therapeutic drug monitoring, noise reduction

## Abstract

SERS, a clinical practice where medical doctors can monitor the drug concentration in biological fluids, has been proposed as a viable approach to therapeutic drug monitoring (TDM) of the antiepileptic drug Perampanel. The adoption of an acidic environment during the SERS experiments was found to be effective in enhancing the spectroscopic signal. In this work, we combine SERS experiments, conducted with a custom spinning cell in controlled acidic conditions, with DFT calculations aimed at investigating the possible protonated forms of Perampanel. The DFT-simulated Raman spectra of protonated Perampanel accounts for most of the observed SERS signals, thus explaining the effective role of protonation of the analyte. Our results suggest protonation as a viable approach to fostering SERS of alkaline drugs.

## 1. Introduction

Surface-enhanced Raman spectroscopy (SERS) is used to detect small traces or even single molecules of analytes by probing their distinctive vibrational signatures. Because of its sensitivity to low-concentration samples and its capability to provide molecular structure-related information, it was considered in many diverse applications [1,2,3,4,5,6,7], down to the single molecule detection limit [8,9,10,11,12]. Among the many possible uses in the biomedical field [13,14], SERS can also be useful in analyzing narrow therapeutic index (NTI) drugs in biological fluids [15]. NTI drugs are difficult to deliver to patients since small variations of concentration in the blood plasma can determine insufficient effects or cause adverse effects [16,17,18,19]. Hence, a clinical practice called therapeutic drug monitoring (TDM) was specifically developed for the treatment of those patients who require NTI drugs. TDM consists of the systematic quantitative analysis of the amount of drug present in the blood plasma, followed by a symptomatic report of the patient treated with the same dosage [17]. Comparing the results of the two analyses usually results in a decrease or increase in the subsequent dosage, until the therapeutic window of the patient is figured out. Antiepileptic drugs (AEDs) are an important group of NTI drugs [18]. Perampanel (PER) is an antiepileptic NTI drug used to treat and prevent partial-onset and generalized seizures that affect, respectively, one or both brain hemispheres [20,21]. The common way to perform TDM is to take a blood sample from the patient treated with NTI drugs to measure the concentration of the drug in blood plasma and then compare the measured value with the response of the patient to accordingly adjust the therapy, thus constructing a response vs. concentration curve that eventually determines the appropriate dose for the patient [17]. The reference analytical procedure currently in use in hospitals is high-performance liquid chromatography (HPLC) coupled with mass spectrometry (MS), which has the disadvantage of requiring extensive treatment of the blood sample, rendering TDM quite costly and time-consuming [22,23,24]. By contrast, SERS may require less involved sample preparation, and the SERS measurement is usually carried out in minutes. These characteristics make SERS a useful technique, complementary to the current analytical TDM techniques, with positive outcomes in the TDM practice [25,26,27,28].

The most common substrates used for SERS are the noble metals Au and Ag, which can enhance the Raman signal thanks to their plasmonic properties, reaching performances comparable to those obtainable with HPLC [29]. In the past, using physical methods, namely, pulsed laser deposition (PLD), we developed spatially uniform SERS sensors made of assemblies of Au nanoparticles on inert supports [15,30,31]. Here, we try a different approach using Turkevich Au colloids [32], which have been widely used for micro-SERS applications for a long time, as well as the closely related Lee–Meisel colloids [33]. Unfortunately, once aggregated in a dried form to promote the SERS action, such noble metal films suffer spatial nonuniformities at the µm scale, at variance with colloidal suspensions that can be profitably used in strictly controlled aggregation conditions [34]. The spatial nonuniformities on the surfaces of the nanostructured films probed by the Raman laser are the culprit for the wide variance of the measurements of SERS intensity at a given analyte concentration, which seriously hinders quantitative analysis [35,36,37,38]. Furthermore, the SERS signal throughput on dried aggregated colloids is also limited by photodamaging, which may occur even at low incident power densities because of the very efficient light-absorbing properties of aggregated colloids.

**Figure 1:**
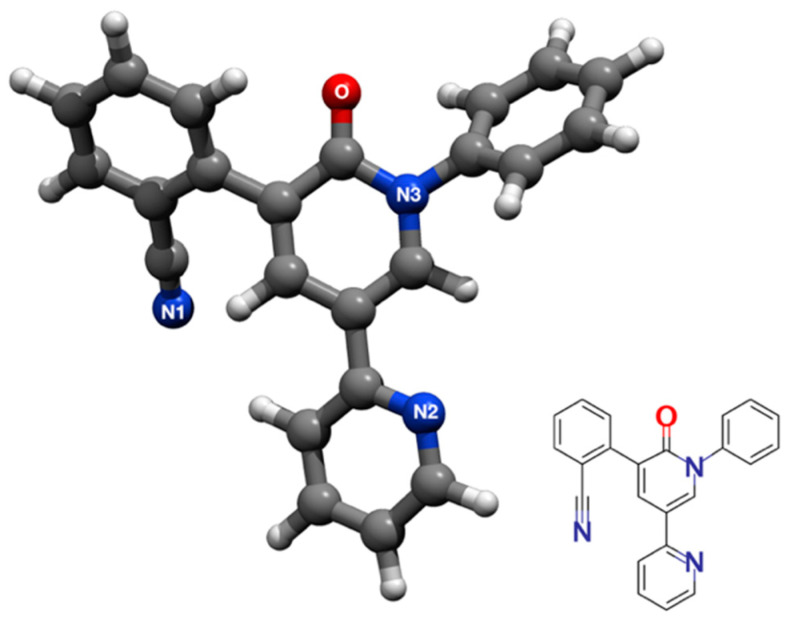
Three-dimensional representation of the lowest energy conformation of the Perampanel molecule (see text for details). The coloring scheme is CPK (C: gray, N: blue; O: red; H: white). The possible proton acceptors are the O, N1, N2, and N3 atoms.

Currently, such two limitations of noble metal colloids pose serious challenges for TDM applications, where one seeks to reliably relate the SERS signal of drugs to their concentration in controlled conditions [15]. Moreover, a recent study on carbamazepine (CBZ) showed that the SERS signal of this AED is fostered by adjusting the pH to acidic conditions [39]. In that study, the authors used HCl, which presumably protonates CBZ. Similarly, we could witness beneficial effects on the SERS response of PER when adopting acidic pH [15,40]. Our observation parallels other works in the literature where the effect of pH on SERS is investigated [41,42]. Since the chemical structure of PER admits several proton acceptor sites (Figure 1), it is relevant to investigate the possible protonated forms of PER, together with the effects of protonation on the vibrational structure of the molecule, which are currently unexplored areas.

The main contributions of this work are the following. First, by DFT calculations, we investigate the possible molecular structures of protonated PER to support the interpretation of the observed SERS spectra of PER in acidic conditions. Second, to improve the quality of the SERS signal obtained using Au colloids, we develop a custom spinning device and rationalize its use by a physical model of its functioning in the micro-Raman spectrometer. Such a device has been obtained by modifying a computer hard drive, which ensures excellent mechanical stability during the rotation at high speed (7200 rpm). We could then use it as a spinning cell to obtain reproducible and high-throughput SERS measurements of PER. The SERS spectra are collected on the spinning disk, allowing a very effective spatial averaging over a large area of the deposited colloids. This spatial averaging during the SERS measurement mitigates the high spatial nonuniformity of aggregated Au colloids. Such an experimental setup also allows for greater laser power, directly increasing the SERS throughput. Indeed, because of the high rotational speed, the residence time of the laser on the focused spot is considerably lowered, effectively preventing photodegradation of the analyte.

## 2. Results and Discussion

### 2.1. The Protonation of PER and Its Effects on SERS

**Figure 2:**
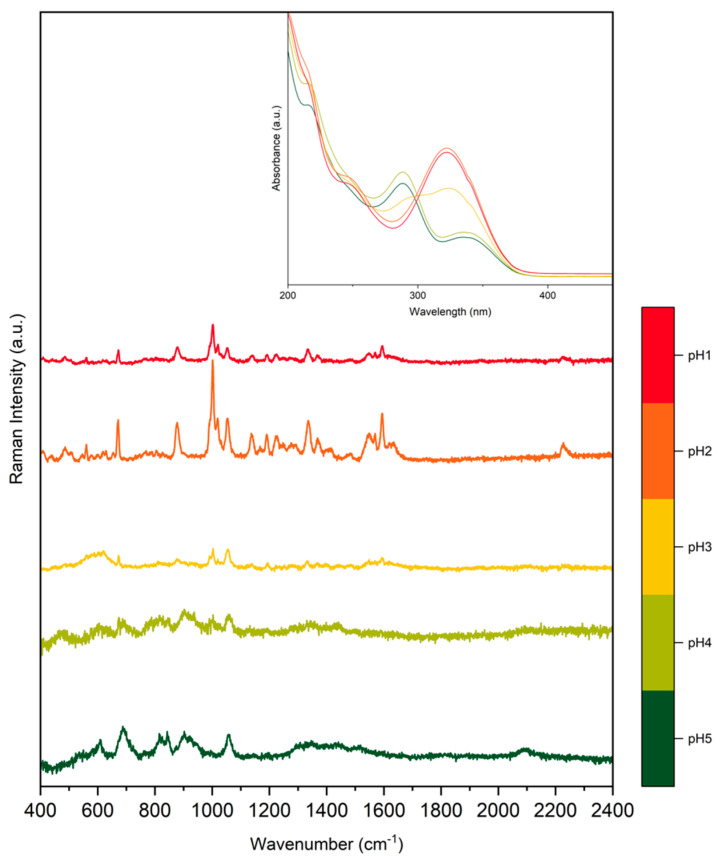
SERS spectra of acidic water solution of PER (5 × 10^−5^ M) at different pH (see text for details). To help the reader appreciate the effect of PER protonation, we also report the UV-Vis spectra of the same PER solutions taken from previous work [13].

When using colloidal Au SERS substrates, the citrate that is present on the surface can hinder the adsorption of some analytes on the metal surface, resulting in SERS spectra where the contribution from the citrate anions may be observed—see for instance ref. [43]. By treating PER with HCl under suitable pH conditions, we may protonate it, and the Cl^−^ ions could also displace the citrate on the surface. This allows for more efficient adsorption of the analyte, which is now protonated and, therefore, more affine to the gold surface, which is clearly decorated with Cl^−^ as proved by the strong AuCl stretching peak observed in the low wavenumber region of the SERS spectra. We have noticed that an excess of Cl^−^ is detrimental to SERS, in agreement with a recent SERS study [44]. One possible solution is to reduce the amount of HCl by substituting a fraction of it with another chlorine-free strong acid. This can be performed effectively by protonating PER in acidic aqueous solutions containing HCl and H_2_SO_4_ with a 1:9 concentration ratio, as described in ref. [15]. The UV-Vis spectra of the PER solutions of such investigation are reported in the inset of Figure 2 as a function of the varying pH. Figure 2 indicates that PER starts to be protonated at pH = 3, and it is fully protonated at pH = 2. We collected the SERS spectra of PER deposited on the substrate from the solutions at different pH values: the results support the hypothesis that acidic environments favor SERS detection. As shown in Figure 2, the SERS spectrum of PER starts to show up at pH = 3 and is best resolved at pH = 2.

**Figure 3:**
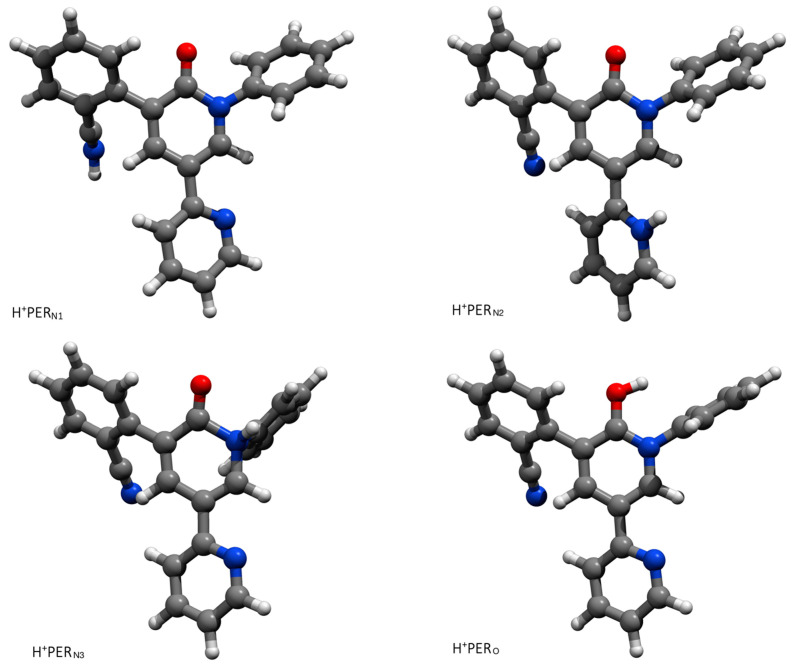
The equilibrium structures of the four protonated forms of PER as determined by DFT (see text for details).

DFT calculations were performed to describe the different protonated forms of PER and assess their possible contribution to the observed SERS spectra of the drug measured in acidic conditions (pH 2). Clearly, the oxygen and nitrogen atoms of PER are identified as the most suitable proton acceptor sites. Thus, four protonated structures are possible, namely, H^+^PER_N1_, H^+^PER_N2_, H^+^PER_N3_, and H^+^PER_O_, whose equilibrium structures are presented in Figure 3. These four structures have been determined by adding a single proton to the equilibrium structure of the lowest energy conformation of PER (see above). Their Cartesian coordinates are reported in the Supplementary Information (Section S3).

Table 1 reports the absolute energy computed by DFT for neutral PER, compared with the energies of the four protonated species. The energy difference ΔE = E^(+)^ − E^(0)^ between the energy of the protonated species, E^(+)^, and the energy of the neutral species, E^(0)^, is a measure of the propensity toward protonation displayed by the different proton acceptors of PER. It turns out that the two strongest proton acceptors are the N2 and O atoms, with an absolute protonation energy |ΔE| that slightly exceeds 240 kcal/mol. By comparison, ammonia (NH_3_), a species well-known for its propensity to easily form the protonated form (ammonium, NH_4_^+^) in the presence of HCl, displays an absolute protonation energy |ΔΕ| of 218.86 kcal/mol (computed with the same DFT method as the data for PER reported in Table 1). These results clearly justify the relevance of all four protonated species of PER in relation to the SERS spectroscopy of PER in acidic conditions.

**Figure 4:**
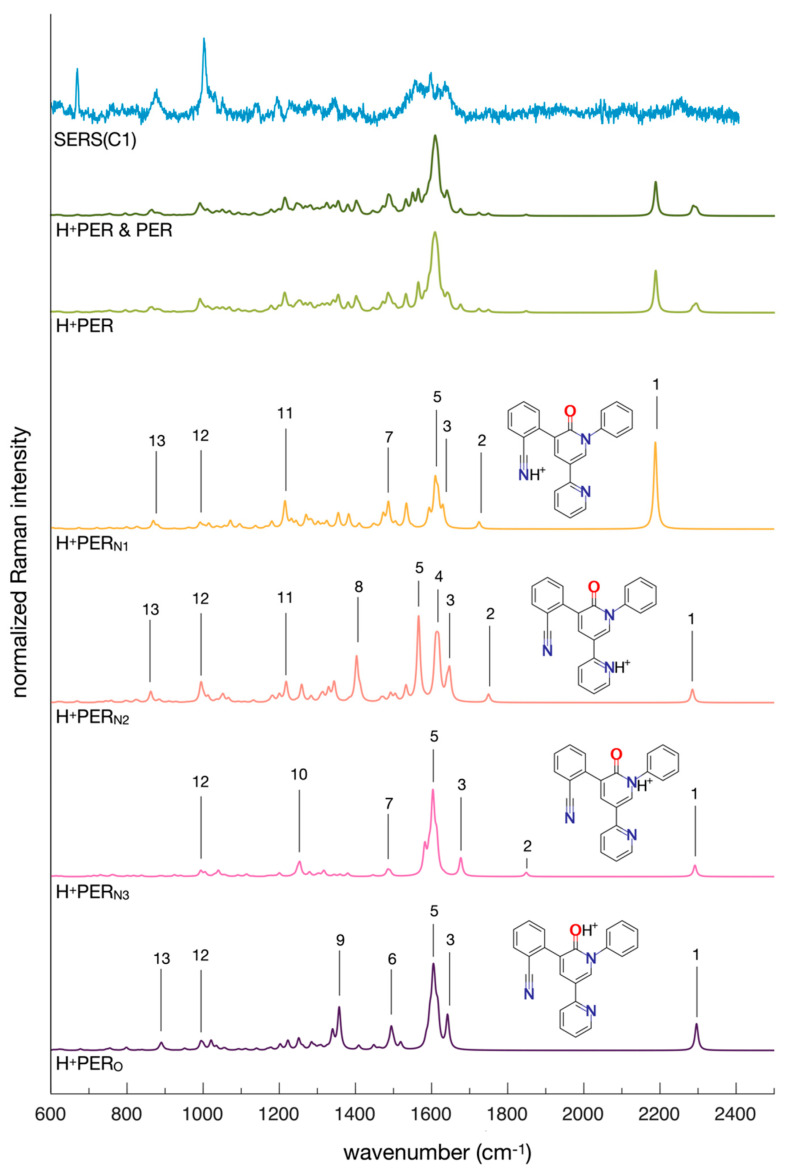
SERS and Raman spectra of Perampanel. From top to bottom: comparison between the results of the experimental dynamic SERS measurements (C1 configuration, see Section 2.2) and the simulated Raman spectra obtained from the sum of the protonated forms of Perampanel (H^+^PER), also including the neutral form (H^+^PER and PER); the spectra of the four protonated forms of Perampanel are also individually reported (H^+^PER_N1_, H^+^PER_N2_, H^+^PER_N3_, and H^+^PER_O_). The wavenumber axis of the simulated spectra has been uniformly scaled by the factor of 0.98 to facilitate the comparison with the experimental SERS spectrum. The peak labels in the figure correspond to the respective assignations in Table 2.

The four spectra of H^+^PER_N1_, H^+^PER_N2_, H^+^PER_N3,_ and H^+^PER_O_ simulated from the Raman intensities and peak positions obtained by DFT calculations are reported at the bottom of Figure 4, whereas their most relevant peaks are assigned in Table 2. Depending on the specific protonation site, the spectra of the four isomers are subject to distinctive modifications. In H^+^PER_N1_ the C≡N triple bond between N_1_ and the adjacent carbon atom has a partial C=N double bond character, which explains the appearance of the intense CN stretching mode at a lower wavenumber, shifted toward the region of C=N double bonds [45] (peak 1). In the spectrum of H^+^PER_N2_, two intense peaks around 1600 cm^−1^ can be observed (assigned to in-plane ring deformation—Table 2), whereas the spectra of the other species exhibit one. By protonating PER at N3 (H^+^PER_N3_), we observe the relative increase in the aromatic ring stretching modes (ca. 1600 cm^−1^), which stand out compared with the other protonated species and the neutral form. Finally, when protonation occurs on the oxygen atom (H^+^PER_O_), the spectrum displays the peak assigned to the stretching of the C-O-H^+^ moiety (peak number 9) instead of the C=O bond stretching peak (peak 2). In summary, the assignment of the simulated spectra of the protonated forms justifies the changes in the molecular structure of PER that are expected because of the interaction with the H^+^ species.

Furthermore, because the propensity for protonation of all four models is significant (Table 1), we have considered a simple algebraic sum of the four simulated Raman spectra as a guideline for the analysis of a representative SERS spectrum of PER recorded at acidic pH (H^+^PER spectrum in Figure 2). The theoretical spectrum of neutral PER was added to this sum as well to account for the possible contribution of non-protonated Perampanel in the acidic solution (H^+^PER and PER spectrum in Figure 2). A wavenumber scale factor of 0.98 was applied to all the theoretical spectra, whereas the baseline of the experimental SERS spectrum was adjusted with the msbackadj function of the Bioinformatics Toolbox [46]. The experimental and simulated spectra qualitatively match: the simulated spectrum correctly indicates the regions where the peaks of protonated Perampanel are present. However, it fails to predict the relative intensities of the peaks. This is due to the very nature of the present computational model, which simply considers isolated protonated PER molecules, whereas the SERS spectrum is generated by protonated PER molecules adsorbed at the gold surface. Another issue is due to the electromagnetic phenomena that occur at the hot spots where SERS originates. In particular, the orientation of the adsorbed molecule with respect to the electric field generated by the plasmon resonance should not be averaged in the same way as for a regular Raman experiment in, e.g., solution state (which is the standard framework of the present simulations), where the Raman intensity is obtained by considering an isotropic orientational average [47].

### 2.2. Testing the Spinning Cell on SERS of PER

**Figure 5:**
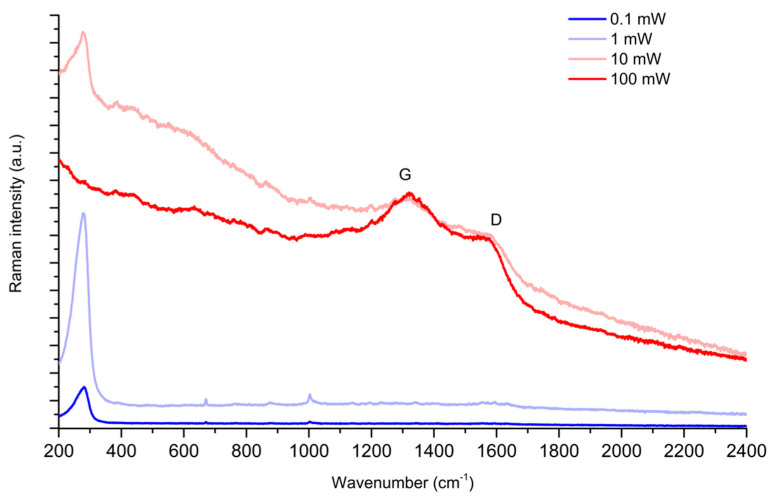
SERS spectra of PER (10^−4^ M, pH2) measured at a fixed position on a Au SERS pad at increasing laser power (785 nm excitation, collected with 0.1, 1, 10, 100 mW laser power, 50x objective). Signatures of damage are evident at a power of 10 mW. The G and D bands arising due to photodamaging of carbonaceous species are highlighted.

To determine a *reproducible* and *high signal-to-noise ratio* SERS spectrum by using dried Au colloids is challenging: strong enhancements are required to get intense spectra, but usually, these come at the expense of a significant spatial inhomogeneity of the dried aggregated colloid. This, in turn, implies two consequences: different local conditions and widely fluctuating signal intensities, which seriously hamper the reproducibility of the SERS measurements. Furthermore, to increase the signal-to-noise ratio, the simplest strategy is, in principle, to increase the laser power. However, the plasmonic enhancement intrinsic to SERS results in a significant sensitivity to laser heating effects that lead to sample degradation, as shown in Figure 5, by the SERS signal of PER collected from a single point for incident laser power increasing from 0.1 mW to 100 mW. Indeed, the SERS spectrum of PER increases in intensity moving from 0.1 mW to 1 mW. However, the first signatures of degradation appear at 10 mW, and at 100 mW, the analyte is fully degraded, as indicated by the typical G and D bands that arise when carbonaceous analytes are photodamaged (Figure 5). We also remark that the peak observed in Figure 5 at 257 cm^−1^, which is assigned to Au-Cl stretching vibrations, is proof of the effective displacement of citrate from the gold surface, promoted by the selected sample preparation procedure. Correspondingly, we do not observe any evident SERS signal that could be attributed to adsorbed citrate species [43].

**Figure 6:**
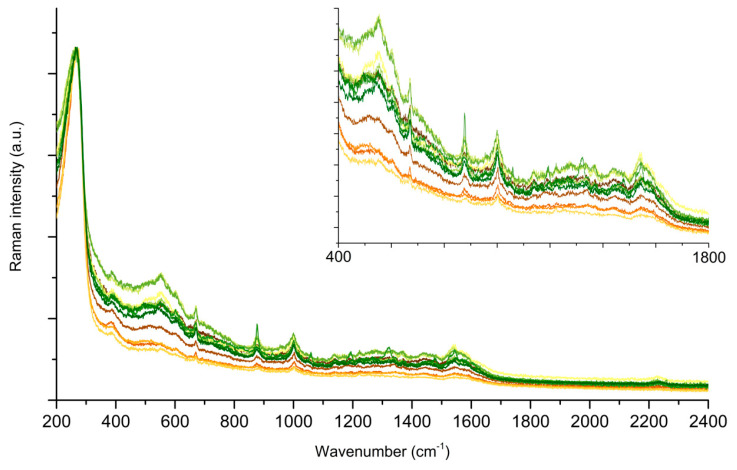
SERS spectra of PER in static mode (10^−4^ M, pH 2). Each measurement is taken at a point spaced 5 µm along a line, with 785 nm excitation, laser power 1 mW, 10 s collection time, 2 averages, and 50x objective. The reported spectra are normalized to the intensity of the AuCl peak at 257 cm^−1^.

In a second series of experiments, we tested the spatial reproducibility of the SERS signal by collecting 12 spectra of PER at different positions of the same Au SERS pad. From Figure 6 the spatial dependence of the signal is sizeable, and there is a great difference in intensity among the spectra collected at different positions.

**Figure 7:**
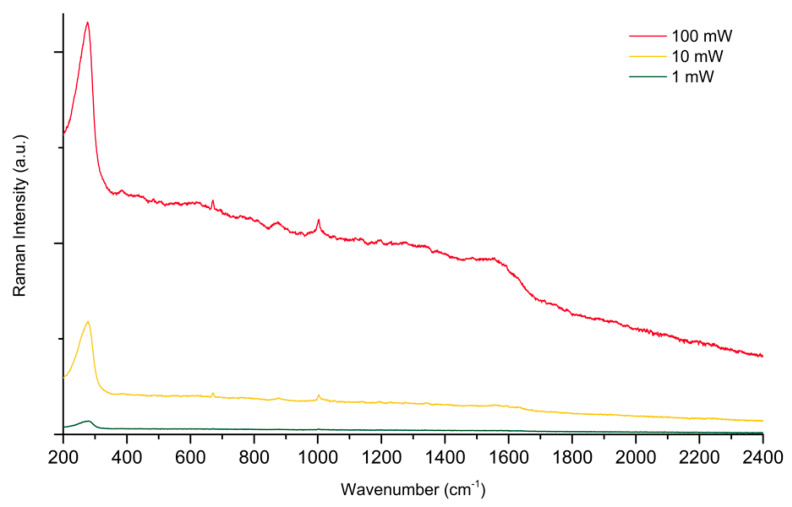
SERS spectra of PER (10^−4^ M, pH 2) at increasing power, with 785 nm excitation. Spectra collected at 1, 10, 100 mW power with C1 configuration. Up to 100 mW of incident laser power, there is no visible sign of photodamaging.

**Figure 8:**
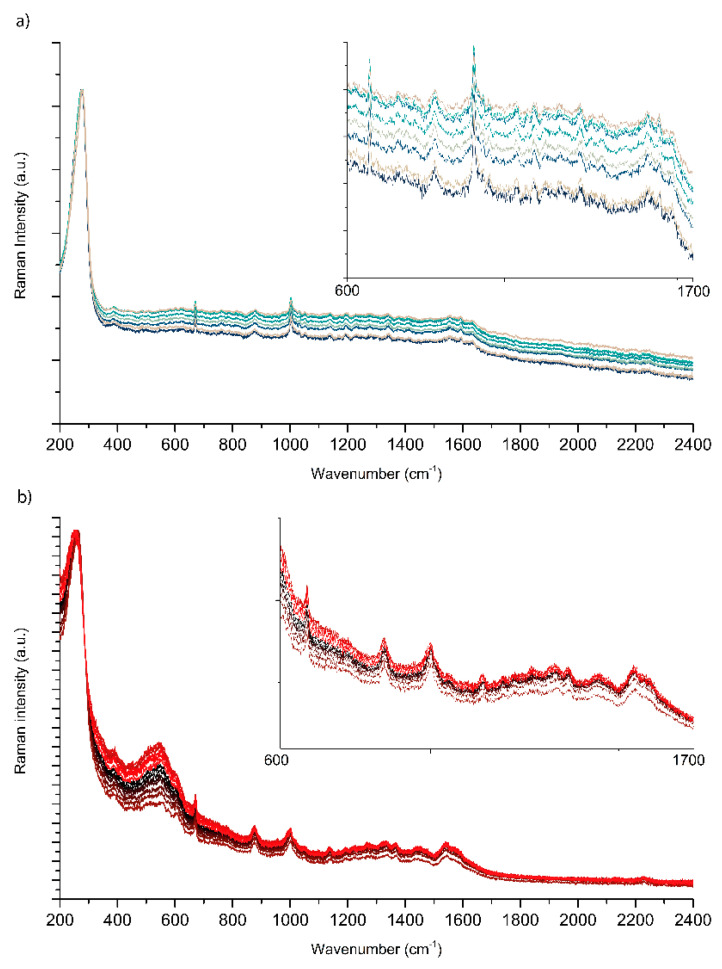
(**a**) SERS spectra of PER in C1 mode (10^−4^ M, pH 2). Each measurement is taken at a point spaced 5 μm axially, with 785 nm excitation, laser power 25 mW, 10 s collection time, and 2 averages. (**b**) SERS spectra of PER in C2 mode. Each measurement is taken at a point spaced 5 µm axially, with 785 nm excitation, laser power 1 mW, 10 s collection time, 2 averages, and 50x objective. The reported spectra are normalized to the intensity of the AuCl peak at 257 cm^−1^.

Hence, the results shown in Figure 7 and Figure 8 prove that by performing static SERS measurements on Au colloidal substrates, we meet dramatic limitations in the maximum laser power that can be used and in reproducibility, due to the inhomogeneous nature of the substrate. This was our main motivation for developing the custom spinning device described in Section 3. As discussed there, by employing a rotating device to perform dynamic SERS measurements, the permanence time τ of the laser on a single point of the substrate is dramatically reduced. Between the two spinning cell configurations considered, C1 appears to reduce τ the most, from the 100 s of the static measurements down to 47 ns. Therefore, we started with collecting the SERS spectra of PER at increasing power in the C1 configuration, with the device set in motion (Figure 7). While increasing the power of the laser from 1 mW to 10 mW, and up to 100 mW, the SERS signal of PER steadily increases in intensity, with no signature of photodegradation (compare for instance the spectrum at 100 mW reported in Figure 7 with that reported in Figure 5).

The second advantage of the spinning device is that by allowing the collection of SERS spectra from different spots located on a circular path, the Raman spectrometer automatically performs the spatial average of the SERS spectra of the analyte as the laser scans through the pad. Moreover, given the small spot size of the laser compared with the scanned area (i.e., given the large η ratio—see Section 3), the spatial average is performed on a very large number of local points. Thus, we expect to significantly increase the spatial reproducibility of the SERS measurements when they are carried out with the spinning cell. To check this hypothesis, we collected eight SERS spectra of PER with the C1 configuration. Each measurement was radially spaced by 5 µm, from an initial distance R = 3.4 cm from the rotation center of the cell. From the first glance at the collected SERS spectra in Figure 8a, the intensity fluctuations are less severe than those observed as a function of the measured spot without the spinning cell (compare Figure 6). Remarkably, despite a small variation in the background, the position and relative intensities of the SERS features of PER are stable and do not display the fluctuations observed without the spinning cell (Figure 6).

Similarly, we collected 12 spectra of PER with the C2 configuration of the spinning cell. Each measurement was radially spaced by 5 µm from an initial distance R = 4 mm from the rotation center of the cell. In this case too (Figure 8b), the reproducibility of the SERS features of PER was good, with modest variations of a featureless background.

To assess the reproducibility of the measurement in static conditions, we carried out a statistical analysis on the intensities of the peaks at 670 cm^−1^ and 1001 cm^−1^ (assigned to coupled ring deformation and out-of-plane C-H bending—see Table 1). The details of this analysis are presented in the Supplementary Information (Section S4). A second analysis was performed in the same way, but the peaks were previously normalized with respect to the height of the AuCl peak. The reason for this choice is that the AuCl intensity depends on the number of chloride anions that are present in the analyte solution deposited on the pads. Because of the procedure in the sample preparation, the Cl^−^ concentration is the same in each measurement (10^−3^ M) and it exceeds the concentration of the analyte. Therefore, we can take the intensity of the AuCl peak as an internal reference in the analysis. In the static mode, the relative error for the non-normalized intensities corresponds to a maximum relative error of 32% for the 670 cm^−1^ peak. Contrary to our expectations, the normalization of the SERS spectra with respect to the AuCl peak does not improve the statistics, and the relative error rises to 48% for the same peak. Most likely, this is due to the very large scatter of the peak heights that we observe when measuring under static conditions. We, therefore, conclude that in this case, we cannot perform reliable quantitative determinations on the analyte concentration.

Considering now the C1 configuration, when the intensities are not normalized, the relative error reaches the maximum value of 11% (for the 1001 cm^−1^ peak), which is significantly lower than the relative error observed with SERS measurement under static conditions (21% for the 1001 cm^−1^ peak). By normalizing the intensities with respect to the AuCl peak, we observe a decrease in the relative error, down to the value of 5% for the peak at 1001 cm^−1^. These results show that using the spinning cell in the SERS measurements significantly improves the reproducibility of the measurements. Finally, an even better scenario is obtained with the C2 spinning configuration. In this case, the relative errors for the non-normalized peak intensities do not exceed 14%, but normalizing the peaks to the intensity of the AuCl peak causes the relative errors to drop to 2.6% for the 1001 cm^−1^ peak and 3.3% for the 670 cm^−1^ peak.

In interpreting the results, one could infer that using an internal reference is the main reason for improving the results, as reported in several studies [48,49,50,51]. However, when considering the effect of normalization, we observe an increase in the relative error in the static measurements, whereas the relative error decreases upon normalization for measurements made with the spinning cell, both in C1 and C2 configurations. We interpret these results as the effect of the wide spatial nonuniformity of the colloid films, which affects the peak of the chloride and of the analyte in a different way, presumably because the chloride promotes aggregation phenomena, whereas the low-concentration analyte does not. Such nonuniform spatial distributions can be effectively averaged out only by the action of the spinning cell. Finally, a remarkable improvement is observed between static and C2 configurations concerning the non-normalized intensities. This indicates that even though normalizing helps to increase reproducibility, the dominant factor in stabilizing the SERS intensities is the dynamic nature of the measurement performed with the spinning cell.

## 3. Materials and Methods

### 3.1. Chemicals

PER was purchased from *Cayman Chemical* (Cayman Europe, Tallin, Estonia; Item No. 23003; CAS 380917-97-5). HAuCl_4_∙3H_2_O, AgNO_3_, trisodium citrate (TSC), and methanol were purchased from Sigma Aldrich and used as received.

### 3.2. Sample Preparation

**Figure 9:**
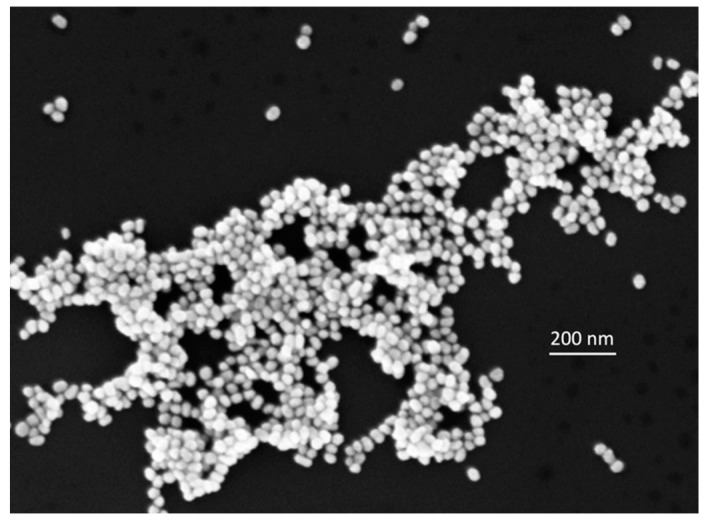
SEM image of the Au nanoparticles deposited on a Si wafer.

(a) PER was first dissolved in methanol to obtain a reference solution at 10^−3^ M concentration. Then, following ref. [15], this reference solution (0.3 mL) was added to an aqueous mixture of HCl and H_2_SO_4_ in a 1:9 molar ratio (2.7 mL) to reach a final pH of 2 at a 10^−4^ M concentration of PER. This allowed us to achieve the protonation of PER without an excess of Cl^-^ that was shown to be detrimental to SERS (see Section 2.1). This relatively high concentration for SERS experiments (10^−4^ M) was selected in such a way as to monitor the evolution of the signal as a function of the tested experimental conditions in the most effective way.
(b) The Au nanoparticles (NPs) were prepared as colloidal suspensions obtained by a modified Turkevich method [32], reducing HAuCl_4_∙3H_2_O with trisodium citrate (TSC). First, in a conical flask, HAuCl_4_∙3H_2_O was dissolved in 180 mL deionized water reaching a concentration of 10^−3^ M, which is about four times higher than the original recipe [32]. Separately, a solution of 1% wt. TSC in deionized water (9.6 mL) was prepared. The two solutions were then heated until boiling, and subsequently, they were mixed in the conical flask. The boiling mixture was kept under magnetic stirring during the formation of the gold NPs for about 60 min. The magnetic stirring was maintained during the cooling of the produced colloidal suspension. Before further use, the colloid was let naturally settle in the conical flask for about one week. To produce the SERS substrates, we withdrew the colloid from the bottom of the flask, where it was more concentrated. A representative SEM image of the gold colloid cast on a Si wafer is shown in Figure 9. The image was acquired in the NanoLab (Energy Dept., Politecnico di Milano) by a Zeiss Supra 40 field-emission scanning electron microscope (FE-SEM), operating in a high vacuum and equipped with the GEMINI column.
(c) We produced SERS-active films (from here on simply denoted as SERS pads) by drop-casting controlled volumes of the Au colloid (20 μL) and letting them dry fully.

### 3.3. Raman Setup

SERS spectra were collected with a dispersive Raman spectrometer (Horiba Jobin Yvon LabRAM HR800, Kyoto, Japan), equipped with a microscope (Olympus BX41, Kyoto, Japan) holding 4×, 10×, 20×, and 50× objectives. We used a 785 nm laser source from a solid-state laser (Laser XTRA, Toptica Photonics, New York, NY, USA) and the 50x objective, which produced a laser spot size of about 1 μm. The Raman spectrometer was equipped with an interference filter to remove spurious lines from the laser. The power at the sample could be modified through a series of filters allowing to adjust the power to 0.1, 1, 10, 25, 75, and 100 mW. When the laser irradiation is backscattered from the sample, an edge filter removes the Rayleigh contribution. The Stokes and anti-Stokes radiations are then dispersed through a grating (600 and 1800 lines/mm). The radiation finally reaches a cooled CCD detector (Peltier) to be accumulated, processed, and visualized. The measurement time and the number of spectra averaged per measurement were controlled via software. The choice of the 785 nm laser was dictated by the use of aggregated Au colloids in the SERS experiments. Because of aggregation, the surface plasmon resonance strongly redshifts (see for instance ref. [52]), and the 785 nm laser approaches resonance conditions.

### 3.4. Spinning Cell

**Figure 10:**
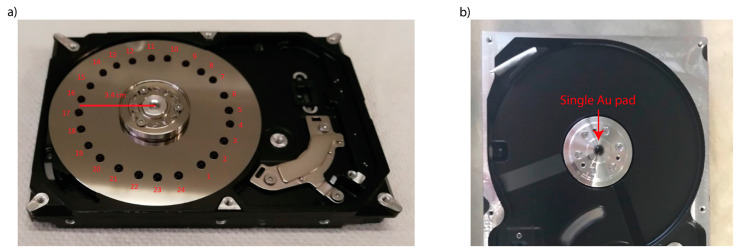
(**a**) Picture of the device in C1 configuration. (**b**) Picture of the device in C2 configuration.

The spinning cell device was obtained from an ordinary computer hard drive used as the mechanical component. The main components of the hard drive were removed, leaving only the rotatory mechanism with the disk. Two configurations were chosen for this work. The first configuration (C1) kept the disk inside the drive. Then we applied 24 SERS pads on the disk along a circumference with a radius of 3.4 cm from the rotation axis. In the second configuration (C2), the disk was removed, leaving the base rotating unit. Then, a single SERS pad was deposited on the top of the rotor, approximately on the axis of rotation. Pictures of the C1 and C2 configurations are shown in Figure 10.

The SERS pads were formed by depositing 20 µL of the Au NP suspension, which produced pads with a diameter in the 3–5 mm range. This choice allowed for furnishing the disk’s surface in the C1 configuration with a reasonable number of pads and a small gap between them. A total of 5 µL of the acidic PER solution was then deposited and let dry on each Au SERS pad. Finally, the spinning device was positioned under the microscope objective of the Raman spectrometer. For the C2 configuration, the laser radiation was focused on a slightly eccentric location on the SERS pad with respect to the rotation axis of the spinning device. The device was then set into motion and the SERS spectra were collected. For both configurations, different spectra were collected by radially displacing the spot of the laser by steps of 5 µm.

During a measurement in the C1 configuration, the focused laser scans through all the SERS pads but inevitably also collects the Raman spectra of the bare surface of the disk between two adjacent pads. C1 allows for the shortest permanence time of the laser on a specific location, minimizing the damage to the pad and the analyte. However, the bare surface of the disk can give a background contribution to the collected SERS spectra, which does not provide peaks that may interfere with those of PER (see Supplementary Information, Section S1). Avoiding this issue would require depositing a continuous ring of colloidal Au NPs on the surface of the disk, thus requiring more of the colloid and more of the sample to be deposited along the ring for the SERS measurement. In the C2 configuration, the laser scans through a circumference within one single pad, which is located close to the rotation axis. Hence, compared with C1, the tangential speed is much lower, so the residence time of the sample under the laser spot is longer. This allows less freedom when choosing the irradiation power but significantly reduces the requirement of colloid and sample volume.

The operation of the spinning device can be described by a simple mathematical model. We take the laser spot diameter as the diameter of the Airy disc produced by an objective of a given numerical aperture (NA) [53]:

(1) $$D=\frac{1.22\;\lambda }{NA},$$

By considering our excitation wavelength (λ= 785 nm) and NA (0.75), we obtain D = 1.2 μm. The laser power per unit area is:

(2) $$P_{S}\;=\;\frac{P}{S},$$

where $S\;=\;\pi D^{2}/4$ is the area of the Airy disc. Using the maximum laser power in our setup, P = 100 mW, we obtain $P_{S}\;=\;8.8\;\times \;10^{7}\;mW/mm^{2\;}$. Under static conditions (i.e., with the spinning device turned off), this laser power density can easily degrade the sample during the total measurement time (τ_SERS_). Typical measurements may require 5 averages of collections of 20 s each, which corresponds to τ_SERS_ = 100 s. With the rotating disk, during the same τ_SERS_, the same optical power is uniformly distributed throughout all the circular ring area scanned by the laser spot $S_{ring}=2\pi RD$, with R >> D being the radius of the ring. Hence, the ratio η by which we effectively increase the probed sample area is:

(3) $$\eta =\frac{S_{ring}}{S}=\frac{2\pi RD}{\frac{\pi D^{2}}{4}}=\frac{8R}{D}=6.56NA\frac{R}{\lambda }$$

Considering the Au SERS pads deposited in the C1 configuration, the average distance between the laser focus and the center of rotation is fixed at R = 3.4 cm. Neglecting the space between adjacent pads, by Equation (3), we estimate a value of η that slightly exceeds 2 × 10^5^. Therefore, P* is the maximum power density allowed in a static SERS measurement to safely avoid photodegradation, and in the spinning cell measurement, we could, in principle, increase the power up to ηP* before reaching the same photodegradation threshold. Since for the C2 configuration, the radius of the ring decreases to R = 4 mm, the value of η correspondingly drops by about one order of magnitude. However, η is still large enough (it exceeds 10^4^) to allow for a significant increase in the laser power during the SERS experiment while safely avoiding photodegradation.

During the operation of the spinning device, the residence time (τ) of the sample under the laser spot can be estimated by the time required by the moving surface, at tangential velocity v = ω R, to travel the laser spot diameter D:

(4) $$\tau =\frac{D}{\omega R}$$

The rotation speed of the hard disk we have employed is 7200 rpm, corresponding to the angular rotational frequency ω = 7.54 × 10^2^ rad/s. From Equation (4) we therefore obtain τ = 47 ns in the C1 configuration, and τ = 0.4 μs, in the C2 configuration.

In both configurations, the permanence time of the laser on a single substrate location is dramatically shorter than the total exposition time for the SERS measure. Thus, in the dynamic mode, the risk of damaging the analyte is considerably reduced, and it becomes possible to adopt higher laser power densities.

### 3.5. Density Functional Theory Calculations

To help the assignment of the main observed spectral features, the Raman spectra of PER were simulated based on the results of DFT calculations carried out with the Gaussian09 program [54]. The vibrational properties of Perampanel were investigated by B3LYP/6-31G(d,p) DFT calculations, and dispersive interactions were accounted for with Grimme’s D3 correction and the Becke–Johnson damping [55,56]. The computed Raman data were used to simulate the spectra as the sum of Lorentzian peaks centered at the computed transition wavenumbers, with intensity given by the computed Raman intensity. In doing that, we assumed a FWHM of 10 cm^−1^ for all the Lorentzian functions.

To determine the molecular structure of PER and its lowest-energy conformer, we have carried out a conformational search workflow driven by metadynamics. To this aim, we used the CREST (Conformer-Rotamer Ensemble Sampling Tool) program [57]. This obtained molecular model of PER, further geometry-optimized by DFT, is validated by the overall good match between the calculated Raman spectrum of such a structure and the experimental Raman spectrum of a Perampanel sample in solid form (powder). The two spectra are reported in Figure S2, and the main peaks are assigned in Table S1 of the Supplementary Information.

## 4. Conclusions

By combining density functional calculations and Raman experiments, we highlighted the direct role of protonated Perampanel species in SERS analyses of acidic solutions of this AED. We showed that using an easy-to-build custom spinning cell improves the quality and reproducibility of the SERS spectra measured on films produced with dried Turkevich Au colloids. We discussed the working principles of the spinning cell with a mathematical model, which justifies the improvements we observed in the SERS signal compared with analogous measurements carried out with static laser-focusing conditions. The results presented here show that combining a spinning cell and protonation is a viable approach for screening and optimizing the conditions for SERS experiments with alkaline molecules. The procedure can be effectively carried out with noble metal Au colloids that can be produced rather straightforwardly. The resulting optimal pH conditions can be later adopted in SERS experiments carried out with other kinds of SERS sensors that are more costly than noble metal colloids.

## Figures and Tables

**Table 1:** Calculated absolute energies in Hartree of neutral PER, H^+^PER_N1_, H^+^PER_N2_, H^+^PER_N3,_ and H^+^PER_O_ and the energy difference between the protonated structures and the neutral molecule. For comparison, the same energy data are also reported for ammonia (NH_3_) and ammonium (NH_4_^+^).

Structure	ΔE (kcal/mol)	E (ha)
PER	//	−1.124985978599593 × 10^3^
H^+^PER_N2_	−244.02	−1.125372595588159 × 10^3^
H^+^PER_O_	−241.30	−1.125368720495620 × 10^3^
H^+^PER_N1_	−216.55	−1.125330688734299 × 10^3^
H^+^PER_N3_	−204.36	−1.125306411724009 × 10^3^
NH_3_	//	−56.55898800030168
NH_4_^+^	−218.86	−56.90775684108217

**Table 2:** Assignment of the most relevant peaks of H^+^PER_N1_, H^+^PER_N2_, H^+^PER_N3,_ and H^+^PER_O_. Label numbers refer to the labels of the corresponding spectrum in Figure 2. The wavenumbers have been uniformly scaled by the factor of 0.98 to facilitate the comparison with the experimental SERS spectrum.

H^+^PER_N1_	Wavenumber (cm^−1^)	Assignment
1	2187	C≡N stretching
2	1724	C=O stretching
3, 5	1629, 1610	in-plane ring deformation
7, 11	1486, 1215	C-N stretching
12,13	993, 868	ring deformation and out-of-plane C-H bending
H^+^PER_N2_		
1	2287	C≡N stretching
2	1746	C=O stretching
3, 4, 5	1649, 1611, 1565	in-plane ring deformation
8, 11	1401, 1219	C-N stretching
12,13	992, 859	ring deformation and out-of-plane C-H bending
H^+^PER_N3_		
1	2292	C≡N stretching
2	1848	C=O stretching
3, 5	1677, 1603	in-plane ring deformation
7	1486	C-N stretching
10	1250	in-plane ring deformation
12	990	in-plane ring deformation
H^+^PER_O_		
1	2296	C≡N stretching
3, 5, 6	1641, 1606, 1486	in-plane ring deformation
9	1251	O-C-N stretching
12	993	ring deformation
13	888	ring deformation and out-of-plane C-H bending

## Data Availability

Not applicable.

## Supplementary Materials

The following supporting information can be downloaded at: https://www.mdpi.com/article/10.3390/molecules28165968/s1, Figure S1. Comparison between the Raman spectrum of the surface of the disk of the spinning-cell (black), and a representative SERS spectrum of Perampanel (red), Figure S2. Comparison between the Raman spectrum of PER simulated by DFT (bottom), and the experimental Raman spectrum of Perampanel in solid form (top). The Raman spectrum was recorded with 785 nm excitation and 20x objective, Table S1. Assignment of the most relevant peaks of the experimental Raman spectrum of PER. The labels identify the Raman peaks in the spectra of Figure S2, Table S2. Coordinates (Å) of the molecular models of Perampanel by DFT of neutral PER and protonated PER: H^+^PER_N1_, H^+^PER_N2_, H^+^PER_N3_, H^+^PER_O_, Table S3. Average peak height (from baseline), standard deviation, and relative error of the SERS measurements in static, C1, and C2 configurations. See the main text for details about the reported normalized data.
