# Supplementary material for: Investigating Perampanel Antiepileptic Drug by DFT Calculations and SERS with Custom Spinning Cell
Source: Molecules. 2023 Aug 9;28(16):5968. doi: 10.3390/molecules28165968 (PMC10459216; doi:10.3390/molecules28165968)
Supplement: Supplementary file 1 [file molecules-28-05968-s001.zip › molecules-2511319-supplementary.pdf]

Supplementary Information

Investigating Perampanel antiepileptic drug by DFT calculations and SERS with custom spinning cell

Nicolò Simone Villa [1,†], Chiara Picarelli [1,†], Federica Iacoe [1], Chiara Giuseppina Zanchi [1] , Paolo M. Ossi [2], Andrea Lucotti [1] and Matteo Tommasini [1,*]

1 Department of Chemistry, Materials, and Chemical Engineering "G. Natta", Politecnico di Milano, Piazza Leonardo da Vinci 32, 20133 Milano, Italy; nicolosimone.villa@polimi.it (N.S.V.); chiara.picarelli@polimi.it (C.P.); chiara.zanchi@tiscali.it (C.G.Z.); andrea.lucotti@polimi.it (A.L.)

2 Department of Energy, Politecnico di Milano, Piazza Leonardo da Vinci 32, 20133 Milano, Italy; paolo.ossi@polimi.it

* Correspondence: matteo.tommasini@polimi.it

† These authors contributed equally to this work.

S1 Raman spectrum of the surface of the disk of the spinning cell

The Raman spectrum of the surface of the disk of the spinning-cell where the SERS pads are deposited in the C1 configuration (see text) is reported in Fig. S1 (black) in comparison with a representative SERS spectrum of PER (red).

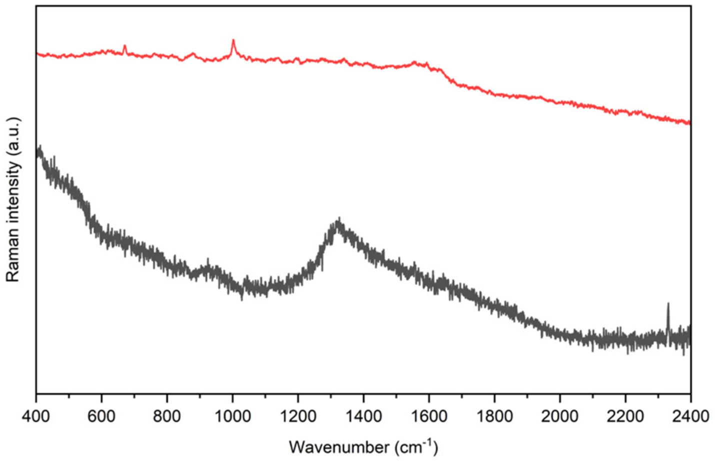

Figure S1. Comparison between the Raman spectrum of the surface of the disk of the spinning-cell (black), and a representative SERS spectrum of Perampanel (red).

S2 Main Raman peaks of Perampanel

The Raman spectrum of Perampanel simulated by DFT methods is shown in Fig. S2 where it is compared with the experimental Raman spectrum recorded on a solid

sample of the drug in powder form (as received from *Cayman Chemical*). The assignment of the main Raman peaks is reported in Tab. S2.

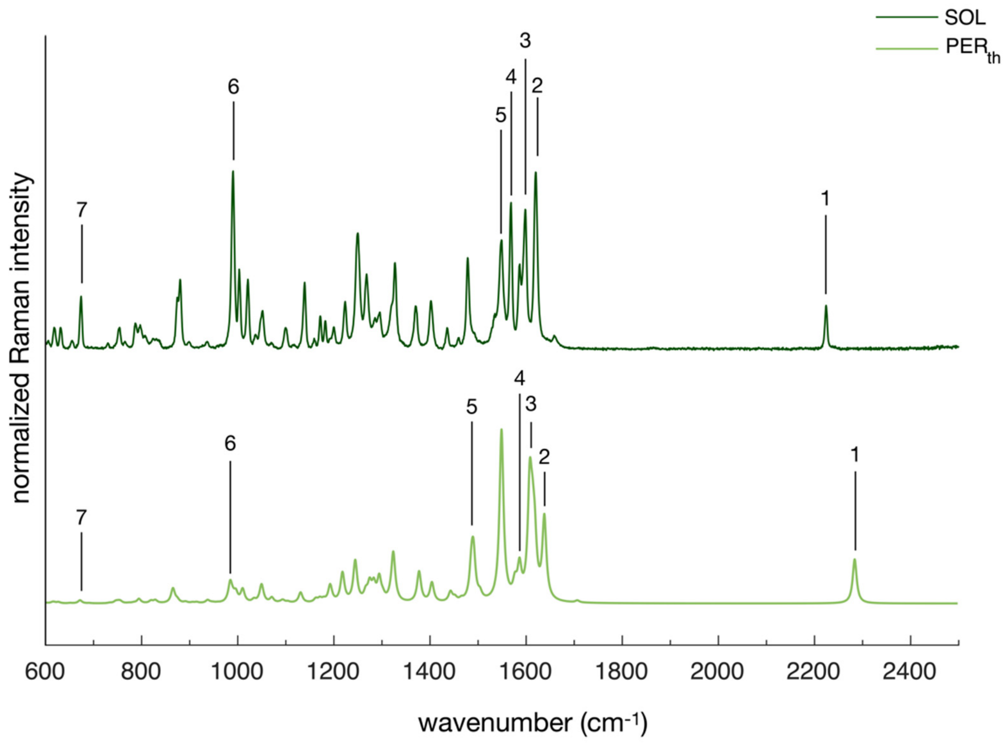

Figure S2. Comparison between the Raman spectrum of PER simulated by DFT (bottom), and the experimental Raman spectrum of Perampanel in solid form (top). The Raman spectrum was recorded with 785 nm excitation and 20x objective.

Label	Expt. wavenumber (cm^{-1})	Assignment
1	2231	C≡N stretching
2, 3, 4, 5	1600, 1563, 1540, 1520	Aromatic ring stretching
6	959	Ring breathing
7	655	Aromatic ring deformation

Table S1. Assignment of the most relevant peaks of the experimental Raman spectrum of PER. The labels identify the Raman peaks in the spectra of Fig. S2.

By applying a scaling factor equal to 0.98 to the wavenumber axis of the calculated spectrum, one obtains a good agreement with the experimental spectrum of Perampanel, notably concerning the C≡N stretching, the aromatic ring stretching modes, and the ring breathing. The aromatic ring deformation mode is present in both spectra, but the predicted Raman intensity is too low compared with the experimental observation.

S3 Coordinates (Å) of the molecular models of Perampanel computed by DFT

Neutral PER

Atom	X	Y	Z
N	1.35499	-0.75459	0.04170
C	0.11046	-1.45797	-0.02531
C	-1.06547	-0.59752	-0.18275
C	-0.91621	0.76623	-0.25961
C	0.34575	1.41059	-0.14806
C	1.44785	0.60616	-0.00961
C	-2.39917	-1.24075	-0.28056
C	-3.55299	-0.68588	0.33470
C	-4.81180	-1.29734	0.19278
C	-4.94702	-2.46409	-0.54830
C	-3.81879	-3.02763	-1.14746
C	-2.56953	-2.42922	-1.01040
C	-3.49762	0.49835	1.14362
N	-3.50237	1.46348	1.79517
O	0.09857	-2.68415	0.03571
C	0.50272	2.88281	-0.16511
C	-0.56222	3.74145	0.15633
C	-0.35190	5.11682	0.11634
C	0.90936	5.60214	-0.22525
C	1.91313	4.67259	-0.50005
N	1.72717	3.35081	-0.47943
C	2.57626	-1.51910	0.13023
C	2.75043	-2.46284	1.14570

C	3.95561	-3.15596	1.23342
C	4.98280	-2.91229	0.31860
C	4.79965	-1.97228	-0.69486
C	3.59362	-1.27780	-0.79561
H	-3.91012	-3.94164	-1.72654
H	-5.92065	-2.93224	-0.65153
H	-5.67054	-0.84735	0.67943
H	-1.70053	-2.88747	-1.46282
H	-1.79186	1.37876	-0.43709
H	2.44033	1.02883	0.07424
H	1.94858	-2.65328	1.84746
H	4.09239	-3.88844	2.02296
H	5.91940	-3.45620	0.39427
H	5.58929	-1.78139	-1.41500
H	3.43665	-0.55894	-1.59368
H	-1.52250	3.34412	0.46754
H	-1.16095	5.79811	0.36278
H	1.11659	6.66624	-0.26768
H	2.91735	5.00751	-0.75466

Protonated PER: H^+PER_{N1}

Atom	X	Y	Z
N	1.290063	-0.851321	0.012331
C	0.004399	-1.466191	-0.128683
C	-1.110811	-0.514212	-0.213915
C	-0.867178	0.850651	-0.255238
C	0.429305	1.390789	-0.153066

C	1.469360	0.489150	0.003471
C	-2.469178	-1.051000	-0.290517
C	-3.569189	-0.413375	0.382797
C	-4.920007	-0.847949	0.216745
C	-5.174565	-1.946376	-0.571700
C	-4.100554	-2.624809	-1.181189
C	-2.785054	-2.199881	-1.035971
C	-3.341996	0.552455	1.346571
N	-3.214774	1.234642	2.295300
O	-0.101030	-2.684049	-0.151304
C	0.733865	2.843874	-0.199102
C	-0.163745	3.787047	-0.720079
C	0.199616	5.132562	-0.720312
C	1.444195	5.498469	-0.213088
C	2.279706	4.487270	0.265869
N	1.941102	3.196086	0.279444
C	2.458389	-1.709166	0.083380
C	2.569619	-2.653850	1.104619
C	3.718424	-3.438455	1.176462
C	4.741758	-3.281383	0.238486
C	4.616187	-2.337880	-0.781083
C	3.468720	-1.549029	-0.865940
H	-4.304518	-3.497760	-1.793410
H	-6.192026	-2.295297	-0.707465
H	-5.719294	-0.327371	0.732723
H	-1.981663	-2.742034	-1.515276
H	-1.704880	1.526842	-0.403438

H	2.484920	0.846215	0.121245
H	1.771168	-2.773694	1.826858
H	3.814327	-4.173053	1.969278
H	5.633369	-3.896818	0.301049
H	5.404682	-2.217588	-1.516680
H	3.353432	-0.827015	-1.668411
H	-1.113283	3.483944	-1.149095
H	-0.475821	5.880685	-1.123767
H	1.767427	6.533429	-0.196901
H	3.265493	4.725554	0.658800
H	-3.097434	2.178231	2.634298

Protonated PER: H^+PER_{N2}

Atom	X	Y	Z
N	1.460508	-0.615411	-0.033513
C	0.291639	-1.467441	-0.116587
C	-0.983237	-0.751246	-0.292187
C	-0.983426	0.609981	-0.409508
C	0.202850	1.398461	-0.275972
C	1.398292	0.732509	-0.083097
C	-2.243207	-1.534569	-0.314926
C	-3.419428	-1.057624	0.329854
C	-4.607588	-1.804568	0.299564
C	-4.649132	-3.025752	-0.365371
C	-3.502108	-3.505257	-0.997873
C	-2.315791	-2.773701	-0.966800
C	-3.431944	0.189158	1.039441

N	-3.434250	1.221271	1.578974
O	0.432557	-2.673015	-0.045574
C	0.108159	2.846760	-0.206822
C	-1.000800	3.526929	0.330461
C	-1.002552	4.911853	0.397482
C	0.100340	5.655342	-0.062601
C	1.172090	4.976058	-0.590062
N	1.141417	3.618868	-0.657597
C	2.757384	-1.244001	0.123431
C	3.015311	-2.051692	1.232330
C	4.282248	-2.612366	1.377761
C	5.277232	-2.370055	0.427094
C	5.005545	-1.567245	-0.680680
C	3.739056	-1.003534	-0.839523
H	-3.525848	-4.457586	-1.517603
H	-5.569447	-3.599689	-0.384219
H	-5.486038	-1.420320	0.806308
H	-1.430567	-3.168760	-1.446908
H	-1.912544	1.123209	-0.619151
H	2.339524	1.239210	0.104235
H	2.237924	-2.240791	1.962812
H	4.491863	-3.240525	2.237209
H	6.260459	-2.813179	0.547199
H	5.771126	-1.388197	-1.428512
H	3.506633	-0.405875	-1.716095
H	-1.828103	2.951862	0.736090
H	-1.856777	5.423263	0.829212

H	0.119445	6.736761	-0.010150
H	2.061342	5.455786	-0.979825
H	1.921221	3.147841	-1.103501

Protonated PER: $H^{+}PER_{N3}$

Atom	X	Y	Z
N	0.211375	-1.620365	-1.172073
C	-1.090865	-0.719067	-1.287009
C	-0.965981	0.600441	-0.683200
C	0.280211	1.080053	-0.406079
C	1.524601	0.340748	-0.571790
C	1.493699	-0.949907	-0.947907
C	-2.206405	1.374595	-0.424992
C	-2.386977	2.035460	0.819961
C	-3.551755	2.775847	1.067595
C	-4.541819	2.866528	0.092599
C	-4.376107	2.213606	-1.128925
C	-3.223277	1.470872	-1.383095
C	-1.398232	1.929139	1.853719
N	-0.572677	1.833752	2.668911
O	-2.001824	-1.246158	-1.852397
C	2.840422	0.992164	-0.324252
C	2.970861	2.126569	0.484741
C	4.247171	2.658865	0.677966
C	5.334142	2.044678	0.065322
C	5.102736	0.901975	-0.709020
N	3.890586	0.382653	-0.903568

C	-0.047234	-2.716566	-0.172829
C	0.073703	-2.435822	1.185422
C	-0.198871	-3.460658	2.091435
C	-0.580333	-4.724718	1.635431
C	-0.698487	-4.975048	0.267538
C	-0.434862	-3.961870	-0.655326
H	-5.145342	2.281298	-1.891038
H	-5.440504	3.441169	0.289511
H	-3.672791	3.266191	2.027130
H	-3.110211	0.969418	-2.336396
H	0.358563	2.103746	-0.062723
H	2.369220	-1.573860	-1.067870
H	0.371222	-1.453622	1.536338
H	-0.110640	-3.266359	3.154866
H	-0.785985	-5.516146	2.348329
H	-0.995269	-5.955935	-0.087597
H	-0.535455	-4.147705	-1.720850
H	2.119450	2.568543	0.990402
H	4.382864	3.534983	1.303594
H	6.340905	2.429920	0.184032
H	5.928166	0.387497	-1.194920
H	0.237358	-2.079454	-2.090357

Protonated PER: H^+PER_O

Atom	X	Y	Z
N	1.270220	-0.777954	-0.028307
C	0.020521	-1.322658	-0.098635

C	-1.105827	-0.487820	-0.248836
C	-0.875180	0.884436	-0.306889
C	0.411934	1.445872	-0.197754
C	1.472480	0.571570	-0.073575
C	-2.479137	-1.056746	-0.319256
C	-3.523301	-0.525131	0.480661
C	-4.821170	-1.051573	0.394627
C	-5.094855	-2.097457	-0.481890
C	-4.074720	-2.623286	-1.275029
C	-2.781157	-2.110014	-1.190292
C	-3.275194	0.551300	1.395887
N	-3.062287	1.442552	2.113272
O	-0.121112	-2.640606	-0.025243
C	0.677275	2.907444	-0.224480
C	-0.310216	3.844821	0.098837
C	0.023432	5.198407	0.051029
C	1.319009	5.562353	-0.305785
C	2.240821	4.549517	-0.586674
N	1.936377	3.250679	-0.549053
C	2.425830	-1.660177	0.118883
C	2.771041	-2.118290	1.393867
C	3.887513	-2.945384	1.527133
C	4.638645	-3.296261	0.403229
C	4.280039	-2.826923	-0.862870
C	3.164699	-2.003137	-1.015929
H	-4.284053	-3.434388	-1.964620
H	-6.100620	-2.498854	-0.544809

H	-5.602234	-0.634887	1.020785
H	-1.999829	-2.526787	-1.815365
H	-1.723524	1.539720	-0.459730
H	2.496900	0.913495	-0.014004
H	2.188308	-1.821617	2.260459
H	4.171920	-3.305690	2.509931
H	5.508055	-3.935757	0.514451
H	4.867724	-3.098836	-1.733117
H	2.875309	-1.627995	-1.992313
H	-1.300133	3.536041	0.417819
H	-0.716945	5.951802	0.299611
H	1.618544	6.603426	-0.356297
H	3.265977	4.793759	-0.854139
H	0.738441	-3.080708	0.104349

Table S2. Coordinates (Å) of the molecular models of Perampanel by DFT of neutral PER and protonated PER: H^+PER_{N1}, H^+PER_{N2}, H^+PER_{N3}, H^+PER_{O}.

S4 Statistical analysis

Configuration	N of Measurements	Peak (cm^{-1})	Average	St.Dev.	Relative Error
**Static**	**12**	670	67.85	21.53	0.317
		1001	102.2	22.00	0.215
**Static (Normalized)**	**12**	670	0.033	0.016	0.476
		1001	0.049	0.019	0.380
**C1**	**8**	670	487.8	38.49	0.079
		1001	359.5	38.22	0.106
**C1 (Normalized)**	**8**	670	0.252	0.024	0.096
		1001	0.185	0.008	0.046
**C2**	**12**	670	82.68	11.41	0.138
		1001	98.66	13.02	0.132
**C2 (Normalized)**	**12**	670	0.053	0.002	0.033
		1001	0.063	0.002	0.026

Table S3. Average peak height (from baseline), standard deviation, and relative error of the SERS measurements in static, C1, and C2 configurations. See the main text for details about the reported normalized data.
